# The prevalence and experience of Australian naturopaths and Western herbalists working within community pharmacies

**DOI:** 10.1186/1472-6882-11-41

**Published:** 2011-05-23

**Authors:** Lesley A Braun, Ondine Spitzer, Evelin Tiralongo, Jenny M Wilkinson, Michael Bailey, Susan Poole, Michael Dooley

**Affiliations:** 1 Cardiothoracic Surgical Research Unit, Department of Surgery, Monash University Alfred Hospital, Melbourne, Australia; 2 Department of Pharmacy, Alfred Hospital, Melbourne, Australia; 3 School of Pharmacy & Griffith Health Institute, Griffith University, Gold Coast, QLD, Australia; 4 School of Biomedical Sciences, Charles Sturt University, Wagga Wagga, Australia; 5 Faculty of Pharmacy and Pharmaceutical Sciences, Monash University, Parkville, Australia; 6 Department of Epidemiology and Preventive Medicine School Public Health & Preventive Medicine, Monash University Alfred Hospital, Melbourne, Australia

## Abstract

**Background:**

Naturopaths and Western herbal medicine (WHM) practitioners were surveyed to identify their extent, experience and roles within the community pharmacy setting and to explore their attitudes to integration of complementary medicine (CM) practitioners within the pharmacy setting.

**Method:**

Practising naturopaths and WHM practitioners were invited to participate in an anonymous, self-administered, on-line survey. Participants were recruited using the mailing lists and websites of CM manufacturers and professional associations.

**Results:**

479 practitioners participated. 24% of respondents (n = 111) reported they had worked in community pharmacy, three-quarters for less than 5 years. Whilst in this role 74% conducted specialist CMs sales, 62% short customer consultations, 52% long consultations in a private room and 51% staff education. This was generally described as a positive learning experience and many appreciated the opportunity to utilise their specialist knowledge in the service of both customers and pharmacy staff. 14% (n = 15) did not enjoy the experience of working in pharmacy at all and suggested pharmacist attitude largely influenced whether the experience was positive or not. Few practitioners were satisfied with the remuneration received. 44% of the total sample provided comment on the issue of integration into pharmacy, with the main concern being the perceived incommensurate paradigms of practice between pharmacy and naturopathy. Of the total sample, 38% reported that they would consider working as a practitioner in retail pharmacy in future.

**Conclusions:**

The level of integration of CM into pharmacy is extending beyond the mere stocking of supplements. Naturopaths and Western Herbalists are becoming utilised in pharmacies

## Background

The term 'integrative (or, sometimes, integrated) medicine' describes a relatively recent development in complementary medicine. It has been defined as practising medicine in a way that selectively incorporates elements of complementary and alternative medicine into comprehensive treatment plans alongside Western medical methods of diagnosis and treatment [1]. The term integrative medicine is not a synonym for complementary medicine but has a larger meaning and mission with a focus on preventative health and healing rather than disease and treatment. In practice, it can be delivered by a single health care provider with appropriate training or relate to health care delivered by a team comprising of orthodox and complementary practitioners working together.

Integrative medicine and the integration of complementary medicine into medical practice have been investigated in a variety of ways in Australia and other countries. Surveys of medical doctors' attitudes, training, usage and referral patterns to complementary medicine practitioners have been documented and models of integrative health care involving medical practitioners have been described [2-8]. In contrast, relatively little has been reported describing the integration of professional complementary medicine services within the pharmacy sector.

Surveys indicate that most Australian adults use complementary medicine products and services usually as an adjunct to their conventional medical care, and often as part of their own self care [9,10]. Community pharmacy indirectly contributes to patients' self-initiated integrative approach as they are a major retail outlet for complementary medicine product sales. Direct contributions to integrative care arise when pharmacists actively recommend holistic and complementary medicine approaches as part of their pharmacy practice.

Advertisements in local papers indicate that Western herbal medicine (WHM) practitioners and naturopaths are practising in some community pharmacies in Australia, thereby giving pharmacy customers an opportunity to receive advice from a person with specialised training in complementary medicine within a pharmacy location. Currently there is a paucity of information describing the interface developing between naturopaths, WHM practitioners and pharmacists and its influence on naturopathic, herbal and pharmacy practice. Little is known about the prevalence of naturopaths and WHM practitioners working in the pharmacy setting, the type of services they provide, their interaction with pharmacists and the attitudes of the broader naturopathic and WHM professions to this emerging model of integrative practice.

The primary aims of this survey were to identify the prevalence, roles and experience of naturopaths and WHM practitioners working within the pharmacy setting. Secondary aims were to explore the attitudes of individual naturopathic and WHM practitioners from the broader naturopathic and Western herbal medicine professions to integration of naturopaths and WHM practitioners within the pharmacy setting.

## Methods

### Questionnaire

A comprehensive anonymous questionnaire was developed in consultation with the National Herbalists Association of Australia and an advisory group consisting of three pharmacists and a consumer advocate. The questionnaire consisted of 37 questions which were adopted and adapted from previous surveys [11-13] and a report published by the Australian National Prescribing Service [14] and included new questions to meet the aims of the study. Thirteen of the 37 questions were specifically related to participants' experiences of working in pharmacy so were only completed by relevant respondents.

The survey instrument was pre-tested by a sample of six herbal and naturopathic practitioners and modified in response to their feedback.

Nine questions collected demographic and workforce information such as gender, age, highest educational qualification in WHM and/or naturopathy, years in practice, place of practice and graduating institution. All survey participants were asked attitudinal questions. Response options varied depending on the type of question asked and included multiple choice, open-ended free text and Likert-scaled responses.

### Data collection

Since there is no national register of naturopaths or WHM practitioners in Australia, random sampling of the population was not possible. Rather a convenience sampling approach was used. Professional associations and specialist complementary medicine product distributors circulated an invitation to practitioners around the country to participate in the study which directed them to the study website, provided project and participant information and a link to the survey questionnaire.

Data collection took place over 5 weeks between March and April 2009. Data were manually entered by participants into SurveyMonkey™, an on-line survey tool. Consent was implied upon agreement to complete the survey questionnaire. Ethics approval was obtained from the Alfred and Monash Human Research Ethics Committee, and subsequently from Charles Sturt and Griffith Universities.

### Data analysis

Descriptive and inferential statistics were calculated using SAS version 9.1 (SAS Institute Inc., Cary, NC, USA). Differences in proportions between groups were compared using chi-square tests for equal proportions or Fishers Exact tests where numbers were small. A two sided p-value of 0.05 was considered to be statistically significant.

## Results

A total of 479 naturopaths and/or WHM practitioners completed the survey (see Additional File 1); a response rate is unable to be calculated as the invitation to participate was distributed both via letter and general email distribution lists. Most (84%) of respondents were female and nearly all (94%) were currently in practice. Table 1 summarises respondents' characteristics.

**Table 1:** Characteristics of survey respondents

		N (%)*
Gender	Male	77 (16)
	Female	399 (84)
	Not reported	3 (1)
Highest level of qualification in naturopathy and/or Western herbal medicine	Certificate	1 (0)
	Advanced diploma	172 (36)
	Undergraduate degree	191 (40)
	Graduate diploma	71 (15)
	Masters degree	19 (4)
	PhD	6 (1)
	Not reported	19 (4)
Year of graduation from naturopathic/ WHM course	2005 - 2009	156 (33)
	2000 - 2004	139 (29)
	1995 - 1999	65 (14)
	1990 - 1994	64 (13)
	1980 - 1989	50 (10)
	Before 1980	5 (1)
Years spent working as a naturopath and/or WHM practitioner	Never	9 (2)
	Less than 1 year	59 (12)
	1-4	145 (30)
	5-9	107 (22)
	10+	150 (31)
	Not reported	9 (2)
Current main place of practice * multiple responses were accepted	Multidisciplinary clinic with other CM practitioners	137 (29)
	Multidisciplinary clinic with medical practitioners	20 (4)
	Naturopathy/herbal medicine clinic as solo practitioner	103 (22)
	Home-based clinic	79 (16)
	In a pharmacy	31 (7)
	In industry (e.g. sales representative)	36 (8)
	Not currently in practice	26 (5)
	Other	90 (19)
	Not reported	16 (3)

*% of total respondents answering question.

A significantly greater number of recent graduates (less than 5 years since graduation) had attained an undergraduate degree qualification rather than an advanced diploma (54% vs. 28%, p < 0.0001) whereas significantly more practitioners that graduated at least 5 years ago had attained a graduate diploma (22% vs. 7%, p < 0.0001) or a masters degree (7% vs. 1%, p = 0.002) compared with more recent graduates.

Of the total sample, 24% (n = 111) reported they had worked in community pharmacy at some time in the past. The majority of these practitioners (75%) had done so for less than 5 years. Eight percent (n = 37) of practising practitioners reported they were currently working in a community pharmacy and 31 practitioners stated it was their main place of work.

### Roles and practice in pharmacy

Naturopaths and WHM practitioners assumed a variety of roles when working in the pharmacy setting, thereby providing multiple responses when asked about their role. Most (81%) conducted specialist CM product sales, 68% conducted short consultations in the pharmacy, 57% conducted long consultations in a private room, 56% provided staff education and 45.5% undertook general product sales. When asked whether they kept their own specialty medicines in the pharmacy, 36% stated they had a large variety of products, 23% a limited range and 25% stated they did not have their own practitioner products in the pharmacy. More specifically, 44% reported they stocked practitioner-only complementary medicine products and 34% had liquid herbal medicines in the pharmacy.

### Information exchange between pharmacists and practitioners

Practitioners working in pharmacy reported that pharmacists referred to them for CM product information (85%), information about other complementary therapies or dietary information (77%) and to provide customer service (77%). In turn, practitioners themselves referred to pharmacists for drug information (93%), safety and drug interaction information (72%) and medical information (55%).

### Experience of working in pharmacy

Of the 111 practitioners reporting they had worked in pharmacy, 100 provided further information about the experience. Many described the experience as providing them with opportunities to learn and be part of a health care team although it was not positive for every respondent (Table 2).

**Table 2:** Respondents description of their pharmacy work experience (n = 100)

Description of experience of working in retail pharmacy	N (%)*
Learnt how to provide advice quickly	59 (59)
Learnt more about over-the-counter CM products	59 (59)
Learnt more about pharmaceutical medicines	58 (58)
Enjoyed being part of health team	47 (47)
Enjoyed working with pharmacist	46 (46)
Focus on sales was problematic	38 (38)
Work was not interesting enough	27 (27)
Did not enjoy it at all	16 (16)
Can't remember	1 (1)
Other	11 (11)

*% of total respondents answering this question (multiple answers possible).

Fifty free-text comments were received which elaborated on practitioners' experiences in pharmacy and re-iterated similar themes to those found in the multiple choice results. Positive comments generally reflected an appreciation for the learning opportunity and team-work environment in pharmacy. Other comments tended to provide further details about practitioners frustration working with pharmacy customers (10 comments), having limited access to CM products which some perceived to be of superior quality to those available over-the-counter in pharmacy (14 comments), limitations of practising in a retail environment (14 comments), perceived lack of respect or support (18 comments) and the importance of pharmacists and/or management attitude on their experience (10 comments).

It appears that naturopaths' experience of working in pharmacies was most greatly influenced by the attitude of the pharmacists (owners or managers). For example,

"[The experience] heavily depends on [the] philosophy of [the] pharmacy owner, [whether they are] supportive of CM or not; purely money motivated to move retail lines; willing or not to use best practise e.g. staff training, appropriate referral, forward[-looking] dispensing practices with customer consultations and thorough case history prior to OTC sale if appropriate, [etc.]".

Two thirds (66%) of the entire sample felt that their undergraduate education had adequately prepared them for real-life practice, however significantly fewer naturopaths and WHM practitioners that had worked in pharmacy considered their education had adequately prepared them for real life practice compared to practitioners that had not worked in pharmacy (59% vs. 70%, p = 0.037).

More than two-thirds of naturopaths (68%) viewed their service in pharmacies as valuable and 20% as somewhat valuable For example,

"I found most clients were extremely grateful to have some realistic advice when confronted with such a daunting choice of complementary products."

Seven percent were unsure how valuable their service in pharmacy was and 2% thought it was not valuable.

Of those that had worked in pharmacy, 99 provided information about the hours worked in this form of employment and 89 responded to questions about wages. Most practitioners reported working more than 15 hours per week in pharmacy, 46% were paid an hourly rate and 42% received a regular wage as a casual or permanent staff member (Table 3). One third found their pay structure satisfactory.

**Table 3:** Income earnings and average number of employed hours in pharmacy work

N (%)*		
Hours/week in pharmacy (h)	Less than 5 hours	5 (5)
	
	5-15	24 (24)
	
	16-24	25 (25)
	
	25-34	17 (17)
	
	35+	28 (28)

Wage (AUD) per hour for pharmacy work	$10-14	11 (12)
	
	$15-19	26 (29)
	
	$20-24	24 (27)
	
	$25-29	14 (16)
	
	$30-34	9 (10)
	
	$35-39	2 (2)
	
	$40+	3 (3)

*% of total respondents answering this question.

Free text comments indicated that some naturopaths did not feel the level of remuneration received was commensurate with their level of education and others raised practical issues about deciding on an appropriate wage. For example,

"Because naturopathy is not registered, my boss had difficulty figuring out how to pay me as there are no standards."

### Attitude of the total sample to pharmacy-complementary medicine integration

Two hundred and eleven people (44%) took the opportunity to provide additional comments in free text about the integration of complementary medicine in pharmacy, indicating significant interest in the topic. Most comments raised concerns about the perceived differences in philosophical paradigms adopted by pharmacists, naturopaths and WHM practitioners and the way the different health care providers understood and managed customers' health care needs. Thirty-five practitioners commented that symptomatic prescribing (assumed to characterise pharmacy practice) was incompatible with naturopathic practice and 35 practitioners raised concerns about the level of knowledge that pharmacists and pharmacy assistants have about CM products.

"I support working together however I do not support or feel comfortable with pharmacists prescribing herbal medicines, just as I should not prescribe pharmaceuticals [etc.]".

Other comments indicated that respondents recognise the demand for over-the-counter complementary medicine products but many do not feel this does justice to the unique, holistic, individualised approach that the naturopathic paradigm and practice offers. One respondent acknowledged:

"There are a lot of grey areas as to how much and what information could and should be given to patients/clients in a retail environment."

Comments indicating potential positive outcomes of integration were also raised, but to a lesser extent. Sixty two respondents proposed that qualified complementary medicine professionals should be available on staff to adequately and safely recommend CMs and some went further by indicating that degree qualifications in naturopathy would represent adequate training. For example:

"I believe the integration of complementary medicines into pharmacy can be beneficial on the basis that a Degree qualified [naturopath or WHM] practitioner is present to oversee and consult with individuals about the remedies they are choosing to ensure members of the public purchase products that are both safe and beneficial to them."

Other comments suggested communication and cross education between pharmacists and CM practitioners may improve with integration (21 comments), customers could receive better, more individualised health care (14 comments) and there would be greater employment opportunities for practitioners (13 comments).

Of the total sample, 57% stated they would not consider working as a practitioner in retail pharmacy in the future. The main reasons cited were perceptions of poor financial rewards, incongruence between the philosophies of naturopathy and pharmacy practice, perceived poor quality CM products available in pharmacies, pressure to sell products, feeling over-qualified for community pharmacy work and lack of respect from pharmacists. One respondent with experience offering naturopathic services in a number of pharmacies suggested:

" [it may be useful to have] a third party helping set up/support the relationship between CM practitioners and pharmacy as naturopaths need to recognise and respect pharmacists' needs to run their business and pharmacists need to understand that naturopathy is more than provision of CM products."

## Discussion

This study identified that naturopaths and WHM practitioners are working within the pharmacy setting, communicating with pharmacists about medicines and complementary health care, providing a variety of customer and staff services and most describe their work as valuable. In over half of instances, naturopathic practice is facilitated on-site with the provision of a private consulting room and, to a slightly lesser extent, practitioners are able to access practitioner-only products. These facts indicate that some level of professional acceptance and integration is occurring within pharmacy and a working model has developed which allows for differences in philosophical beliefs about health and wellbeing to co-exist within the one location. Comments received from practitioners that have worked in pharmacy describe an 'uneasy truce' whereby naturopaths recognise they gain new knowledge, skills and the experience of working in a health care team when in pharmacy, yet some feel frustrated by pharmacists' attitudes and with the limitations of providing a retail service, and many are dissatisfied with the remuneration received.

Practitioners from the broader naturopathic and Western medicine profession who participated in the study hold concerns about reconciling the differences in health care philosophies between pharmacists and naturopaths however many consider it important for qualified complementary medicine professionals to be available on staff to adequately and safely recommend CMs in the pharmacy setting.

Although this study did not explore the reasons why some pharmacists employ naturopaths, it can be assumed they recognise that naturopaths and WHM practitioners are providing added value to their pharmacy's services. Increasing product sales is one likely motivation however another may relate to pharmacists themselves feeling ill-prepared to respond to customer queries about complementary medicine products. Surveys have shown that consumers expect pharmacists to be knowledgeable about complementary medicine products [15] and most pharmacists consider it important to have complementary medicine knowledge and be able to provide patient information [16-18] however pharmacists generally rate their complementary medicine knowledge as inadequate and are not confident in answering patient enquiries [12,17-19].

### Financial considerations for naturopaths and Western herbalists

Naturopaths and WHM practitioners in private practice have two main income streams, consultation fees and product sales [13]. They are also responsible for paying the expenses associated with running a small business such as rent and utilities, staff wages and stock. Within the pharmacy setting, we identified that most practitioners worked more than 15 hours per week and received either an hourly rate or a regular wage which, for the majority, was higher than the award rate for a pharmacy assistant but lower than for a pharmacist.

Although most respondents are dissatisfied with their wage, it is possible that working in pharmacy affords a steady income for practitioners unable or unwilling to set up their own private practice and may be viewed as a 'style of internship in health care, something that is lacking in the current career paths of many complementary medicine professionals'[20].

### Integrative medicine in the pharmacy?

Integrated health care has been described in the literature using various conceptual models. In general, they develop around four key components: philosophy and values, organisational structure (e.g. roles and hierarchy), process (e.g. communication and decision making methods) and outcomes (e.g. physical, mental and overall wellbeing) [21]. Another conceptual framework describes seven different integrated models which encompass individual practice, integrative group practice and practice integration within institutional settings such as hospitals and universities [22].

Our results indicate that naturopaths, WHM practitioners and pharmacists that work at the same location appear to provide professional services in parallel. Sometimes naturopaths and WHM practitioners work in the public retail area alongside pharmacy assistants whereas in other locations they conduct private consultations in a separate consulting room and have access to practitioner-only products. Whilst there is some discussion between naturopaths and pharmacists about different medications and safety issues, it is unclear whether they share information in a consultative or collaborative way about specific customers.

Previous discussions about integrative health care models have tended to downplay the tensions inherent in different paradigms of health care practice and the problems that arise when the philosophies of complementary medicine and biomedicine co-exist. This study has clearly identified several practical problems and professional concerns from the complementary medicine perspective, as different ad hoc models of naturopathic integration within the pharmacy setting have developed.

### Towards a better model

The results of this study provide much needed information to the naturopathic, WHM and pharmacy communities which may benefit from each others expertise in the community pharmacy setting. Currently pharmacists and naturopaths must individually negotiate work arrangements resulting in ad hoc models of integration developing. Patient care and the professional practices of both pharmacists and complementary medicine practitioners would benefit from a more directed approach. Ideally, guidelines should be developed through a collaborative consultative process between key stakeholder groups to promote patient care and safety and which takes into account issues such as inter-professional communication, defining scope of practice, when to refer, award rates for naturopaths and WHM practitioners and professional indemnity. The development and evaluation of a new integrative model involving naturopaths and WHM practitioners that also takes into account the quality use of medicines principles, as described in the national medicines policy, should be the subject of further study. Future research into the nature of the information exchange between naturopaths and pharmacists would also be worthwhile.

Like all studies, the interpretation of our findings is limited by several possible sources of bias. As there is currently no centralised national registration board of naturopaths and WHM practitioners, it was impossible to determine a response rate or ensure all eligible practitioners received information about the study, We did not collect data about non-responders although our responder characteristics of gender and age are similar to two other previous Australian surveys of naturopaths and WHM practitioners [11,13]. We also did not collect data about practitioner location so comparisons to other surveys in this regard are not possible. Future research could address this issue. The effects of self-report, self-selection and recall bias are also unknown and highlight a need for future research to confirm our findings.

## Conclusion

The level of integration of complementary medicine into pharmacy is extending beyond the mere stocking of supplements. Naturopaths and Western Herbalists are becoming utilised in pharmacies and providing customers with shop floor advice and private consultations and even providing staff education. Whilst many of the naturopaths and Western herbalists that had worked in pharmacy found the experience rewarding, the practical and philosophical issues raised in this study need to be resolved before naturopaths and Western herbalists more fully explore this form of integration.

## Competing interests

Authors of this manuscript have the following to disclose concerning possible financial or personal relationships with commercial entities that may have a direct or indirect interest in the subject matter of this presentation:

Dr Lesley Braun is Vice President of the National Herbalists Association of Australia.

There is nothing else to disclose for all authors.

## Authors' contributions

LB conceived of the study, was project manager, co-ordinated data collection and chief author of this paper. ET and JW contributed to study design, co-ordinated data collection at their sites, MB was chiefly responsible for statistical analysis, OS, SP and MD aided in study design and all aided in results interpretation and have read and approved the final manuscript.

## Pre-publication history

The pre-publication history for this paper can be accessed here:

http://www.biomedcentral.com/1472-6882/11/41/prepub

## Supplementary Material

Additional file 1 **Survey of naturopaths and western herbal medicine practitioners**. This is the complete survey used to collect data from study participants. Most results are presented in this article whereas others form the basis of a second paper to be published separately. Click here for file
