# Supplementary material for: The prevalence and experience of Australian naturopaths and Western herbalists working within community pharmacies
Source: BMC Complement Altern Med. 2011 May 23;11:41. doi: 10.1186/1472-6882-11-41 (PMC3128856; doi:10.1186/1472-6882-11-41)
Supplement: Additional file 1 — Survey of naturopaths and western herbal medicine practitioners. This is the complete survey used to collect data from study participants. Most results are presented in this article whereas others form the basis of a second paper to be published separately. [file 1472-6882-11-41-S1.PDF]

Herbalist and Naturopath Survey

1. INVITATION TO PARTICIPATE IN A NATIONAL SURVEY

welcome page

THE INTEGRATION OF COMPLEMENTARY AND ALTERNATIVE MEDICINES INTO COMMUNITY PHARMACY PRACTICE - A NATIONAL NATUROPATH AND HERBALIST SURVEY

What is the aim of this research?
This is a new phase of a larger study which is investigating the integration of complementary and alternative medicines into community pharmacy.

It has been initiated because results from our customer survey, which involved over 1000 people, identified a substantial interest in having greater access to naturopathic and/or herbal medicine practitioners. As a naturopath and/or herbalist, we are interested in your attitudes, behaviours, knowledge and perceptions so that we can explore this area further. We will not be collecting personal information such as your name thereby ensuring complete confidentiality.

Benefits of participation
The inclusion of this new phase into the study provides the naturopathic and herbal medicine professions an opportunity to improve inter-professional communication and promote a better understanding.

What are we asking from you?
You are invited to participate in the study by completing this survey. Your agreement to participate is assumed if you complete the survey.

How long does the survey take to complete?
It will take approximately 10 minutes of your time. Participation is voluntary and you will be able to exit the survey at any time. Most questions in the survey ask you to tick a box to indicate your response.

How will the information from this survey be used?
The results will be used to develop methods of aiding pharmacists in the delivery of quality use of medicines strategies. Results will also be made available to participating associations which may aid them in future planning.

They will also be used for publication in professional journals, reports and newsletters and presented at conferences. Information will be stored in a secure location at the Alfred Hospital as per hospital policy.

Further information can be found at: http://www.alfred.org.au/camstudy/

Who is responsible for this survey?
This survey is part of a larger study being conducted by Professor Michael Dooley, Dr Lesley Braun, Susan Poole (Monash University / Alfred Hospital), Dr Evelin Tiralongo (Griffith University) and Dr Jenny Wilkinson (Charles Sturt University). The study is funded by the Department of Health and Ageing and the Pharmacy Guild of Australia.
The Human Research Ethics Committee of the Alfred Hospital, Melbourne, Victoria has given approval for the research.

If you have any complaints about any aspect of the project, the way it is being conducted or have any questions about being a participant, then you may contact: Ms Rowan Frew, Ethics Manager, Alfred Research and Ethics Unit. Telephone: (03) 9076 3848

Your participation in this research is important and very much appreciated!

2. Section One

Information About You

Herbalist and Naturopath Survey

1. What is your gender?

- Male
- Female

2. What is your age?

3. What is the postcode of the premises of your main place of work?

4. What is your highest level of qualification in naturopathy/herbal medicine?

- Certificate
- Advanced diploma
- Undergraduate degree
- Graduate diploma
- Masters
- PhD

Comments

5. What is the name of your undergraduate naturopathy/herbal medicine training institution?

6. What year did you graduate?

7. Do you feel your undergraduate training has adequately prepared you for 'real life' practice?

- Yes
- No

Please provide further comment:

8. Have you trained in any other modality?

- No
- Yes

If yes, please specify:

9. Which of the following best describes your main place of practice?

- Naturopathy/herbal medicine clinic as solo practitioner
- Multidisciplinary clinic with other complementary medicine practitioners
- Multidisciplinary clinic with medical practitioners
- Home-based clinic
- In a pharmacy
- In industry (e.g. sales representative)
- Not currently in practice
- Other

Other (please specify)

10. How long have you worked as a herbalist/naturopath?

- I have never worked as one
- Less than 1 year
- 1-4 years
- 5-9 years
- 10+ years

3.

11. Have you ever worked as a herbalist/naturopath in a community retail pharmacy?

- Yes
- No

4.

12. How long have you worked in the pharmacy sector?

Herbalist and Naturopath Survey

13. Are you currently working in a community retail pharmacy?

- Yes
- No

14. On average, how many hours per week do/did you work in a pharmacy?

15. What is/was your role in the pharmacy? (Mark all that apply)

- General shop/floor sales (all pharmacy products)
- Specialist complementary medicine product sales
- Quick consultations (on shop floor)
- Long consultations (in private room)
- Providing staff education
- Other

Other (please specify)

16. Do/did you stock you own complementary medicines (CMs) in the pharmacy? (Mark all that apply)

- Yes - a large variety
- Yes - a limited range
- Yes - practitioner only CM products
- Yes - liquid herbal medicines
- No
- Other (please specify)

Other (please specify)

17. Do/did you refer your clients anywhere else to purchase complementary medicines?

- No
- Yes

If yes, please provide reasons

Herbalist and Naturopath Survey

18. How would you describe your experience of working in a retail pharmacy? (Mark all that apply)

- I do/did not enjoy it at all
- I enjoyed being part of a health team
- I enjoyed working with a pharmacist
- I found the work was not interesting enough
- The focus on sales was problematic for me
- I learnt more about pharmaceutical medicines
- I learnt more about over-the-counter complementary medicine products
- I learnt how to provide advice quickly
- I can't remember
- other(please specify)

additional comments

19. How are/were you paid?

- A regular weekly wage (permanent or casual staff)
- An hourly rate (amount varies depending on hours worked)
- On commission of sales

If on commission, please indicate approximate percentage:

20. For a typical week, what is/was your approximate income from retail pharmacy work?

21. Do/did you find this pay structure satisfactory?

- Yes
- No

If no, what would you find satisfactory?

22. Does/did the pharmacist refer to you for the following? (Mark all that apply)

- Complementary medicine (CM) product information
- Other CM information (e.g. therapies, diet information)
- To provide customer service
- None of the above
- Other (please specify)

Other (please specify)

23. Have you ever asked the pharmacist for the following? (Mark all that apply)

- Drug information
- Medical information
- Safety and interaction information
- None of the above
- Other (please specify)

Other (please specify)

5.

24. How would you describe the service you provide/provided in-store?

- Valuable
- Somewhat valuable
- I'm not sure how valuable
- Not valuable
- Other comments

Other comments (please specify)

6.

Herbalist and Naturopath Survey

25. Do you currently work as a herbalist / naturopath in any of the following locations?

- Health food store
- Medical clinic
- Educational institution
- None of the above

7.

26. Would you ever consider working as a herbalist/naturopath in a retail pharmacy?

- Yes (please provide more information below)
- No (please provide more information below)
- Not applicable

Please provide more information:

27. Have you ever contacted a pharmacist to discuss the following? (Mark all that apply)

- Drug interaction information
- Drug safety information
- General drug enquiries
- Medical information (e.g. disease information)
- None of the above
- Other (please specify)

Other (please specify)

Herbalist and Naturopath Survey

28. Have you ever contacted a general practitioner or other medical practitioner to discuss the following about a patient? (Mark all that apply)

- Drug interaction information
- Drug safety information
- General drug enquiries
- Medical information (e.g. disease information)
- None of the above
- Other (please specify)

Other (please specify)

8. Section Two

Your Knowledge About Complementary Medicine Products

29. Which of the following complementary medicines induce a clinically significant interaction with the listed medications? (Mark as many as apply, using the drop down boxes)

	Warfarin	Digoxin	Oral contraceptive	Oral hypoglycaemic
St John's wort				
Ginkgo biloba				
Magnesium				
Echinacea				
Fish oils				

30. Which of the following complementary medicines have clinically proven benefits for the listed indications? (Mark as many as apply, using the drop down boxes)

	Osteoarthritis	Upper respiratory tract infections	Diarrhoea	Cardiovascular disease	No clinically proven benefit
Fish oils					
Glucosamine sulphate					
Probiotics					
Echinacea					
Coenzyme Q10					

9. Section Three

Information Sources

Herbalist and Naturopath Survey

31. What are the 3 main information sources you use for complementary medicine products?

- I don't refer to information sources
- World Wide Web in general e.g. Google
- Databases such as Pubmed/Medline
- Specific website/s
- Drug reference texts e.g. MIMs
- Medical reference texts
- Complementary medicine textbooks
- Peer-reviewed medical journals
- Complementary medicine journals
- Manufacturer literature, seminars, and/or representatives
- Professional seminars, conferences or lectures
- Naturopath/herbalist colleagues
- Other (please specify)

Other (please specify)

32. If you have a favourite resource, please provide details below.

33. What are the 3 most important factors you consider when assessing information about complementary medicine products?

- It is available online or on my desktop (computer)
- It is available as a textbook or in hard copy
- It is available at no cost (e.g. manufacturer literature)
- The information has a scientific basis
- It contains both traditional and scientific information
- It contains information about a wide range of complementary medicines - those with evidence and those without
- It is not produced by manufacturing companies
- It is updated frequently
- It is an Australian information source
- Other (please specify)

Other (please specify)

Herbalist and Naturopath Survey

34. How important do you consider each of the following as sources of evidence about complementary medicine products?

	ESSENTIAL	IMPORTANT	UNSURE	NOT IMPORTANT	OF NO VALUE
Randomised controlled clinical trials with humans					
Animal studies					
Traditional/historical use well documented					
Epidemiological (population) studies					
Published case studies					
Colleague recommendation					
Personal experience					
Patient reports and feedback					

35. Do you have any other comments about the integration of complementary medicines into pharmacy?

36. Please check one box to indicate your opinion about the following statement. A comments box is provided if you want to add extra information.

	Strongly agree	Agree	Unsure	Disagree	Strongly disagree
Naturopaths and Western herbalists should be formally registered to safeguard the public					

Other (please specify)

37. Thank you for participating.

Please tell us how many minutes this survey took you to complete.
